# 520 Are Predicted Protein Recommendation Adequate When Unable to Utilize Nitrogen Balance Testing?

**DOI:** 10.1093/jbcr/irae036.155

**Published:** 2024-04-17

**Authors:** Lacey Patton, Scott W Mueller, Arek J Wiktor, Cameron Gibson

**Affiliations:** University of Colorado Hospital, Littleton, CO; UCHealth and University of Colorado School of Pharmacy, Aurora, CO; University of Colorado Hospital, Aurora, CO; University of Colorado, Aurora, CO; University of Colorado Hospital, Littleton, CO; UCHealth and University of Colorado School of Pharmacy, Aurora, CO; University of Colorado Hospital, Aurora, CO; University of Colorado, Aurora, CO; University of Colorado Hospital, Littleton, CO; UCHealth and University of Colorado School of Pharmacy, Aurora, CO; University of Colorado Hospital, Aurora, CO; University of Colorado, Aurora, CO; University of Colorado Hospital, Littleton, CO; UCHealth and University of Colorado School of Pharmacy, Aurora, CO; University of Colorado Hospital, Aurora, CO; University of Colorado, Aurora, CO

## Abstract

**Introduction:**

Evidence based practice on providing adequate protein for burn patients is limited. Recommendations range from 20-25% of overall caloric needs deriving from protein, or 1.5 – 4 grams (g) per kilogram (kg) of body weight. Our American Burn Association (ABA) verified burn center uses both indirect calorimetry (IC) and urine urea nitrogen balance (UUN) as a guide for patient specific caloric and protein needs. The objective of this study was to evaluate protein requirements based on 25% of predicted IC caloric needs compared to urine UUN.

**Methods:**

This was a single center retrospective analysis at an ABA verified burn center. We included patients with 10% or greater total body surface area (TBSA) burns between 3/15/23 and 9/1/23 who completed an IC and a UUN. We excluded occurrences of invalid IC results, inaccurate protein intake records, or if a UUN occurred >7 days from the IC. Expected (Exp) protein requirements were calculated by 25% of IC resting energy expenditure multiplied by 1.3 activity factor. Observed (Obs) protein requirements were calculated by a collected 12-hour UUN + 4 grams for a ne positive nitrogen balance. Correlation of Exp vs Obs protein and matched mean difference with an assessment of estimate bias was conducted overall and as subgroups based on TBSA 10-19.9 and 20% or greater.

**Results:**

After excluding 28 incomplete assessment pairs, 15 patients representing 34 IC and UUN assessments (8 with >1 assessment) were included. Median age and TBSA were 31 years (30, 52.5) and 20% (13.6, 33.1), respectively. Total cohort Exp and Obs protein requirements were 198 and 157 g (matched mean difference 41 g, p< 0.001), and did not correlate (Pearsons coefficient 0.06, p=0.73). In those 20% TBSA or greater, 8 patients (median TBSA 31.1%; 21.7, 59) had 23 assessments. Exp vs Obs protein requirements were 205 and 160 g (matched mean difference 45 g, p=0.001) and, poorly correlated (Pearsons coefficient 0.11, p=0.62). In those 10-19.9% TBSA, 7 patients (median TBSA 12.75%; 10.8, 14.75) had 11 assessments. Exp vs Obs protein requirements were 184.5 and 150.5 g (matched mean difference 34 g, p=0.073) and, weakly correlated (Pearsons coefficient 0.22, p=0.51).

**Conclusions:**

Calculating protein demands based on 25% of IC caloric requirements may overestimate protein needs when compared to a measured UUN. Larger studies and continued research in nutrition support are needed to improve accuracy of estimated protein requirements.

**Applicability of Research to Practice:**

In patients with large burns (>10% TBSA), burn centers should consider using UUN to more accurately determine patients’ adequate protein needs.

